# Modeling the Optical Properties of a Polyvinyl Alcohol-Based Composite Using a Particle Swarm Optimized Support Vector Regression Algorithm

**DOI:** 10.3390/polym13162697

**Published:** 2021-08-12

**Authors:** Taoreed O. Owolabi, Mohd Amiruddin Abd Rahman

**Affiliations:** 1 Physics and Electronics Department, Adekunle Ajasin University, Akungba Akoko, Ondo 342111, Nigeria; taoreed.owolabi@aaua.edu.ng; 2 Department of Physics, Faculty of Science, Universiti Putra Malaysia, Serdang 43400, Malaysia

**Keywords:** polyvinyl alcohol, composite, support vector regression, refractive index, particle swarm optimization, energy gap

## Abstract

We developed particle swarm optimization-based support vector regression (PSVR) and ordinary linear regression (OLR) models for estimating the refractive index (n) and energy gap (E) of a polyvinyl alcohol composite. The n-PSVR model, which can estimate the refractive index of a polyvinyl alcohol composite using the energy gap as a descriptor, performed better than the n-OLR model in terms of root mean square error (RMSE) and mean absolute error (MAE) metrics. The E-PSVR model, which can predict the energy gap of a polyvinyl alcohol composite using its refractive index descriptor, outperformed the E-OLR model, which uses similar descriptor based on several performance measuring metrics. The n-PSVR and E-PSVR models were used to investigate the influences of sodium-based dysprosium oxide and benzoxazinone derivatives on the energy gaps of a polyvinyl alcohol polymer composite. The results agreed well with the measured values. The models had low mean absolute percentage errors after validation with external data. The precision demonstrated by these predictive models will enhance the tailoring of the optical properties of polyvinyl alcohol composites for the desired applications. Costs and experimental difficulties will be reduced.

## 1. Introduction

Polyvinyl alcohol is an atactic, semi-crystalline polymeric material that possesses excellent biodegradability, biocompatibility, useful mechanical properties, excellent optical properties, and non-toxicity, hence its wide range of applications [1,2,3]. Other excellent properties of polyvinyl alcohol include thermal stability, water solubility, excellent optical transmission, and non-corrosiveness [4]. These features, especially its optical properties such as the refractive index and energy gap, promote its industrial and technological uses as an optoelectronic material, a coating material, a solar cell component, a super capacitor component, and a component of several kinds of sensors [5,6]. The hydrogen bonding between polyvinyl alcohol and other materials is facilitated by the presence of hydroxyl groups on the carbon backbone of polyvinyl alcohol, and these bonds help with composite formation [6,7]. Polyvinyl alcohol is of significant interest because it is abundantly accessible, relatively cheap, contains many volatile functional groups, and has hydrophilic features. It has excellent charge storing capacity, has great dielectric strength, and gives uniform high-optical-quality films for nonlinear optical instruments and optical sensors. Temperature dependency and inter or intramolecular connectivity enhance polyvinyl alcohol chains’ flexibility [8]. These properties strengthen the polyvinyl alcohol matrix, making it a viable composite that can be used for electronic devices, bioengineering, and optoelectronics. Fillers have been incorporated as dopants to modify and tailor polyvinyl alcohol’s optical properties for specific applications [8].

A polymeric composite involves reinforcement and the incorporation of a filler into the parent polymer matrix, which ultimately results in enhanced physical, optical, electrical, chemical, and mechanical properties [9]. The properties of these polymeric composites are strongly influenced by the nature of the parent polymer; the concentration of the filler; the mutual interaction between the polymer and the filler; and the size, shape, and type of the modified composite [8]. Fillers take different forms, including metal powders, carbon fiber, chalk, volcanic minerals, glass fibers, polymeric fibers, and natural fibers. The hydroxyl groups on the carbon backbone of polyvinyl alcohol eases composite formation with other materials and provides viable ways of enhancing its optical properties (refractive index and energy gap). The refractive index is one of any polymer’s most important optical features, due to its influence on and connection with electrical, optical, and magnetic properties. Adequate knowledge of the refractive indices of polymers is important for their applications in waveguide manufacturing, optical fibers, and optical films, among others [10]. Polyvinyl alcohol compounds with high refractive indices are highly desirable in photonics and optics, due to their potential to increase light output by reducing reflection losses [11].

We developed particle swarm-based support vector regression (PSVR) for predicting the refractive indices of polyvinyl alcohol composites while using the energy gap as a descriptor. Filler incorporation alters the energy gap of any polyvinyl alcohol due to trap-level formation within the band gap when an impurity is incorporated into the polymer matrix [12]. Linear relations exist between the energy gaps and the refractive indices of semiconductors [13]. The refractive indices of heterogeneous polymers have been predicted in other ways [10]. We utilized the aforementioned relation by developing machine learning-based predictive models for estimating the refractive indices and energy gaps of atactic polyvinyl alcohol composites for the first time, due to the importance of polyvinyl alcohol for technological advancement. Ordinary linear regression (OLR)-based models were also developed in this work to demonstrate nonlinear relationship between the refractive indices and energy gaps of polyvinyl alcohol composites, and to further clarify the inadequacy of linear models in addressing the relationship.

Support vector regression (SVR) belongs to the category of nonlinear intelligent algorithms. It operates according to the principle of structural risk minimization, using statistical learning [14]. It addresses nonlinear problems conveniently through nonlinear mapping functions and efficiently handles small samples of datasets with excellent predictive outputs [15,16]. The algorithm has enjoyed wider applicability lately due to its robust mathematical computation and global convergence feature [17,18,19,20,21,22]. The hyperparameters associated with SVR include the epsilon, penalty factor, kernel function, and kernel parameter. Accurate determination of the hyperparameters of the algorithm remains the key to the precision of the model. Tuning of these hyperparameters is often achieved through manual searching, grid searching, or the use of heuristic algorithms. Among the heuristic algorithms, the particle swarm optimization (PSO) algorithm has demonstrated a high rate of success and excellent processing time [23,24]. It avoids local convergence and attains fast convergence rate without doing so prematurely. Excellent features of PSO were combined with those of SVR to develop PSVR-based models so that the optical properties of polyvinyl alcohol composites could be estimated with high precision. We developed hybrid intelligent models for determining the influences of fillers or dopants on the refractive index (n) and energy gap (E) of polyvinyl alcohol using particle swarm-based support vector regression (PSVR) algorithms. The performances of the developed hybrid intelligent models were compared with those of ordinary linear regression (OLR)-based models using various performance metrics.

The content of the remainder of this manuscript is as follows. Section two presents the mathematical formulation of SVR and PSO algorithms. Section three describes the computational details of the hybridized algorithms, physical descriptions of the materials, and the acquisition of the implemented dataset. Section four explains the results of the models and presents a comparison with the outcomes of the ordinary linear regression models. Section five concludes the manuscript.

## 2. Mathematical Descriptions of the Algorithms

The formulation of the support vector regression algorithm is mathematically described in this section. The evolutionary principle governing particle swarm optimization is also presented.

### 2.1. Support Vector Regression

Support vector regression can connect the energy gap of doped polyvinyl alcohol with the corresponding refractive indices through data transformation from a two-dimensional structure to a higher-level structure of n dimensions [25,26]. Consider a dataset of M samples of polyvinyl alcohol composite consisting of input energy gap $E_{k}\in X={\mathbb{R}}^{m}$ and measured refractive indices $n_{k}\in Y=\mathbb{R},$ such that k=1,2,…,M. The algorithm addresses the problem through a regression function presented in Equation (1) [27,28].

(1) n(E)=〈γ•E〉+b

where γ and b are vector weights and bias, respectively, where γ,b∈ℝ. The dot product between the input E and weight vector γ is represented by 〈γ•E〉. Restricting the precision of the model to a threshold value defined by epsilon ε requires that the Euclidean norm shown in Equation (2) is minimized and subjected to the constraints and conditions of Equation (3) [29,30].

(2) $$min\frac{{\left\|\gamma \right\|}^{2}}{2}$$

(3) $${\{\begin{array}{c} n^{meas}-\langle \gamma •E_{k}\rangle -b\leq \varepsilon \\ \langle \gamma •E_{k}\rangle +b-n^{meas}\leq \varepsilon \end{array}}_{\;}$$

where measured and estimated refractive indices are denoted by $n^{meas}(E)$ and n(E), respectively. Positive variables (χ and $\chi^{*}$) known as slack variables penalize the prediction function in situations where the precision threshold defined by ε becomes difficult to actualize. With these inclusions, the optimization problem is transformed to Equation (4), and the new constraints contained in Equation (5) hold.

(4) $$Min\frac{{\left\|\gamma \right\|}^{2}}{2}+C\sum_{k=1}^{M}\left(\chi^{*}+\chi \right)$$

(5) $$\{\begin{array}{c} n^{meas}-\langle \gamma •E_{k}\rangle -b\leq \varepsilon +\chi \\ \langle \gamma •E_{k}\rangle +b-n^{meas}\leq \varepsilon +\chi^{*} \end{array},\chi^{*},\chi \geq 0_{\;}$$

where C is the penalty coefficient that influences the precision and accuracy of the model. It penalizes samples outside the channel through determination of the tradeoff between model complexity and the training error. A small value of C indicates a regression function permitting a lower-cost deviation of the predicted refractive index from the measured values. Thus, the epsilon loss function is defined in Equation (6).

(6) $${\left|n^{meas}-n\right|}_{\varepsilon }=\{\begin{array}{c} 0\;,\;\left|\chi \right|\leq \varepsilon \\ \left|\chi \right|-\varepsilon ,otherwise \end{array}$$

It should be noted that the refractive indices of the trained sample of polyvinyl alcohol composites, which fall within negative and positive ε zone, do not fall within the loss. Lagrange multipliers are adequate for solving the convex optimization problem contained in Equation (4). Lagrange multipliers $\left(\lambda ,\lambda^{*},\delta ,\delta^{*}\right)$ are introduced as presented in Equation (7).

(7) $$\begin{array}{ll} L(b,\gamma ,\lambda ,\lambda^{*},\chi^{*},\chi ,\delta ,\delta^{*}) & =Min\frac{{\left\|\gamma \right\|}^{2}}{2}+C\sum_{k=1}^{M}\left(\chi_{k}{}^{*}+\chi_{k}\right)-\sum_{k=1}^{M}\delta_{k}\chi_{k}-\sum_{k=1}^{M}\delta_{k}^{*}\chi_{k}^{*}\\ & \;+\sum_{k=1}^{M}\lambda_{k}\left(n(E_{k})-n_{k}^{meas}-\varepsilon -\chi_{k}\right)+\\ & \;\sum_{k=1}^{M}\lambda_{k}^{*}\left(n(E_{k})-n_{k}^{meas}-\varepsilon -\chi_{k}^{*}\right) \end{array}$$

The final regression function after Lagrange multipliers and subsequent transformation to original dual space is presented in Equation (8) [31].

(8) $$n(E)=\sum_{k=1}^{M}\left(\lambda_{k}-\lambda_{k}^{*}\right)E_{k}•E+b$$

The support vectors acquired during the training phase of model development with the training samples correspond to $\lambda_{k}-\lambda_{k}^{*}\neq 0$. These support vectors represent the data points which are closer to the hyperplane and can influence the orientation and the position of the hyperplane. Inclusion of kernel function $\eta \left(E_{k},E\right)$ into Equation (7) allows nonlinear mapping, and the new regression function is presented in Equation (9) [32]

(9) $$n(E)=\sum_{k=1}^{M}\left(\lambda_{k}-\lambda_{k}^{*}\right)\eta \left(E_{k},E\right)+b$$

The Gaussian kernel function presented in Equation (10) performs better than the other functions. This kernel is a robust radial basis kernel which has excellent anti-interference defense against data noise.

(10) $$\eta \left(E_{k},E\right)=\exp\left(-\frac{{\left\|E_{k}-E\right\|}^{2}}{\omega }\right)$$

where ω is the kernel parameter.

### 2.2. Particle Swarm Optimization (PSO)

Particle swarm optimization is a metaheuristics-based method of optimization that was inspired by fish training and bird swarming. The algorithm addresses optimization problems by considering a flock of birds with social interactions among themselves in a search for sources of food [33,34]. Each bird searching for food sources is considered a particle; the swarm refers to the flock of birds. Velocity and position are two characteristic features that direct the swarm towards the food sources, and these features are determined randomly at the initial stage of the search. When a bird attains an ideal position, the position is referred to as its individual best, since the position factors in the peculiarities of the bird itself. However, the global best position comes into play when a bird attains the best position with respect to the swarm [31,35]. With the individual experience of each bird and the experiences perceived by other birds in the swarm, the position and velocity (individual best and global best positions and the velocities) are updated and refreshed accordingly. The position and velocity of each of the particles (bird) are mathematically modeled and simulated as shown in Equations (11) and (12), respectively.

(11) $$R_{j}(i+1)=R_{j}(i)+V_{j}*\left(i+1\right)$$

(12) Vj(i+1)=(ψ∗(Vj(i))+(ac∗τ∗(Pj−Rj(i))+(ac^∗τ^∗(Pj^−Rj(i))) 

where $R_{j}$ = jth particle position (N-dimensional), $V_{j}$ = jth particle velocity (N-dimensional), ψ = weight (inertial), $a_{c}$ = first acceleration constant, τ = random number in a range of 0 to 1, $P_{j}$ = individual best position, ac^ = second acceleration constant, τ^ = another random number from 0 to 1 (it may be different or the same as τ), and Pj^ = global best position. The inertial weight controls the stopping conditions of the algorithm and decreases as the number of iterations increases [36]. The relation with which the inertial weight controls the convergence of the algorithm is presented in Equation (13).

(13) $$\psi =\psi_{\max}-\left(\frac{\psi_{\max}-\psi_{\min}}{i_{\max}}\right)i$$

where $i_{\max}$ and i, respectively, represent the maximum number of iterations defined by the user at the commencement of the algorithm, two hundred, and the number of iterations at a particular time.

## 3. Hybrid Particle Swarm-Based Support Vector Regression Model Development

The computational part of this work is presented in this section. Data acquisition and a description of the dataset are also presented.

### 3.1. A Description of the Dataset and Its Acquisition

**Figure 1:**
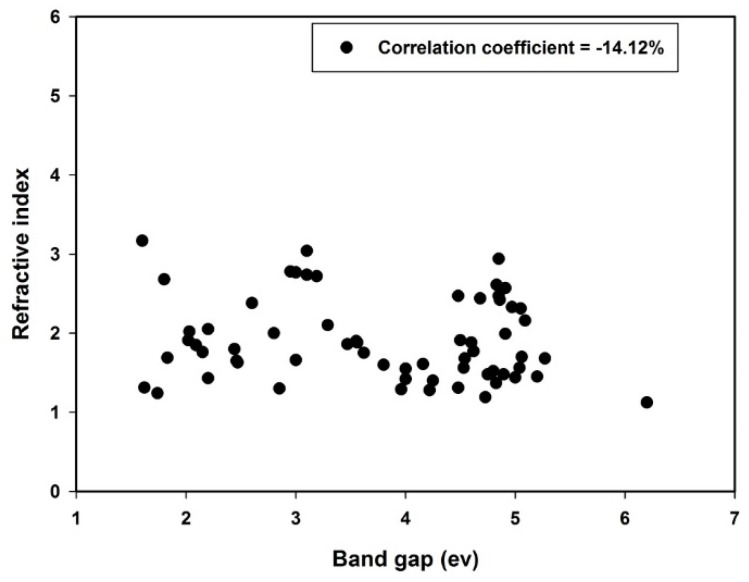
Correlation cross-plot between the descriptor and target.

The refractive indices and energy gaps of polyvinyl alcohol composites used for developing PSVR and OLR models were extracted from the literature [6,7,12,37,38,39,40,41,42,43,44,45,46]. The energy gaps and refractive indices dataset was extracted from sixty-three composite samples of polyvinyl alcohol. Increments in the concentrations of fillers within the polymer matrices influence the refractive indices of the polymer composites due to crosslink formation in the respective matrices. The refractive index of a polymer changes with the density of the crosslinking because of the tightness and closeness between the chains [6]. Similarly, impurities (fillers) incorporated into the polymer matrix lead to trap-level formation within the band gap, which consequently affects the energy gap of the composite [12]. The correlation cross-plot between the refractive indices and energy gaps of polyvinyl alcohol composites is presented in Figure 1. It can be inferred from the figure that there exists no linear relationship between refractive index and energy gap for the investigated polymeric composites.

### 3.2. Computational Methodology of the Particle Swarm Optimized Support Vector Regression

Development of PSVR and OLR-based models was conducted within the MATLAB computing environment. The hyperparameters influencing the precision, robustness, and accuracy of support vector regression were optimized using a particle swarm optimization algorithm, in which each bird (particle) in a flock (swarm) was assumed to contain the information about the hyperparameters in a specified order. The dataset employed for the simulation was randomized before proceeding to the data partitioning phase to ensure uniform distribution of the data points. The randomized set of data was further separated into training and testing at 8:2. The training set was employed for support vector acquisition. The effectiveness and efficacy of each model were assessed using the testing set. The step-by-step procedures of the algorithm hybridization are summarized as follows:

**Step 1:** Particle swarm parameter and search space initialization: PSO parameters such as the population size (N_P_), maximum number of iteration ($i_{\max}$), inertial weight (ψ), and acceleration constants (ac^ and $a_{c}$) were initiated and specified. The search spaces for each of the hyperparameters were also defined as [1000 1; 0.9 0.1; 0.9 0.01] for the E-PSVR model, corresponding to [penalty factor, epsilon, kernel option]. The search space for the n-PSVR model was defined as [1000 1; 0.9 0.1; 0.9 0.1], corresponding to [penalty factor, epsilon, kernel option]. It should be noted that the limits of the search spaces were selected after performing a random check of the most probable locations of the possible solutions.

**Step 2:** Random generation and initialization of particle position and velocity: The position and velocity of each of the particles constituting a swarm were generated randomly within the search space. The generated position and velocity were potential values of the hyperparameters.

**Step 3:** Fitness function evaluation: Evaluation of the fitness of each of the particles involves the development of an SVR-based model by implementing the following major steps. (i) Selection of a function (such as sigmoid, polynomial, or Gaussian) which serves as the kernel function. (ii) The selected function, a particle from a swarm, and the training data are incorporated into the SVR algorithm to train a model. (iii) The trained model is evaluated using root mean square error (TR-RMSE). (iv) The testing dataset is fed into the support vectors acquired during the training for model validation. (v) The tested model is also evaluated using root mean square error (TS-RMSE). Therefore, each particle within the swarm has the corresponding value of TS-TMSE (that is, individual best $P_{j}$) which serves as the fitness value. The lower the value of TS-RMSE, the fitter the particle. When the lowest value (corresponding to the most fit particle) of TS-RMSE in a swarm is compared with the lowest values of TS-RMSE of the other swarms, the lowest TS-RMSE from all the swarms is referred to as the global best (Pj^).

**Step 4:** Updating the individual best positions: If the value of the particle current position ($P_{current}$) is greater than $P_{j}$, update the position as $P_{current}=P_{j}$. Otherwise, proceed to the next step.

**Step 5:** Global position update: If Pcurrent>Pj^, update as Pcurrent=Pj^. Otherwise, proceed to the next step.

**Step 6:** Iteration continuation: If a particle’s index is greater than the initially defined number of particles, proceed to the next step. Otherwise go back to **Step 3**.

**Step 7:** Fitness evaluation using global best position: Using the global best position, evaluate the fitness function of the particles.

**Step 8:** Velocity and position update: Update the velocity and position of the particle using Equations (11) and (12), respectively.

**Step 9:** Stopping conditions: The algorithm stops the repeating circle if the maximum number of iterations has been attained. Otherwise, go to **Step 2**.

**Figure 2:**
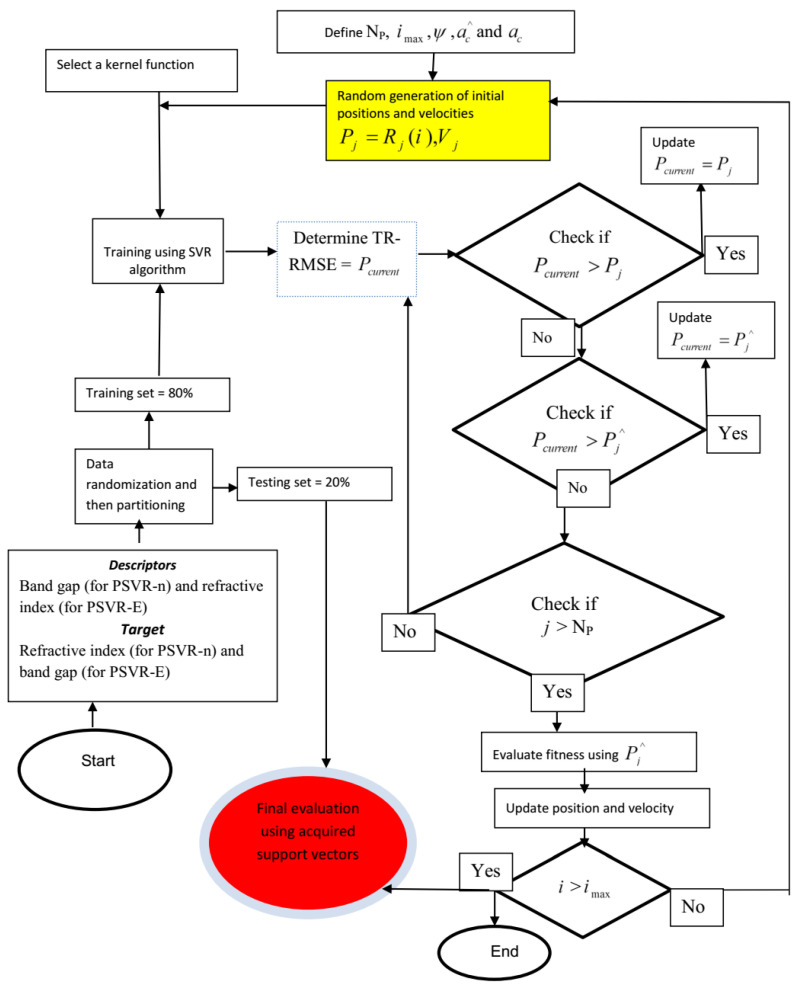
Computational architecture of the developed PSVR-based models.

The computational flow description of the developed PSVR-based models is presented in Figure 2. The complete code is available at the Supplementary Materials.

## 4. Results and Discussion

The outcomes of the developed n-PSVR, n-OLR, E-PSVR, and E-OLR models are presented in this section. The dependencies of the developed models on the number of particles in the swarm are presented. Results of the investigation of the influences of fillers on the optical properties of polyvinyl alcohol composite are also presented.

### 4.1. Convergence and Sensitivity of the Developed PSVR-Based Models

**Figure 3:**
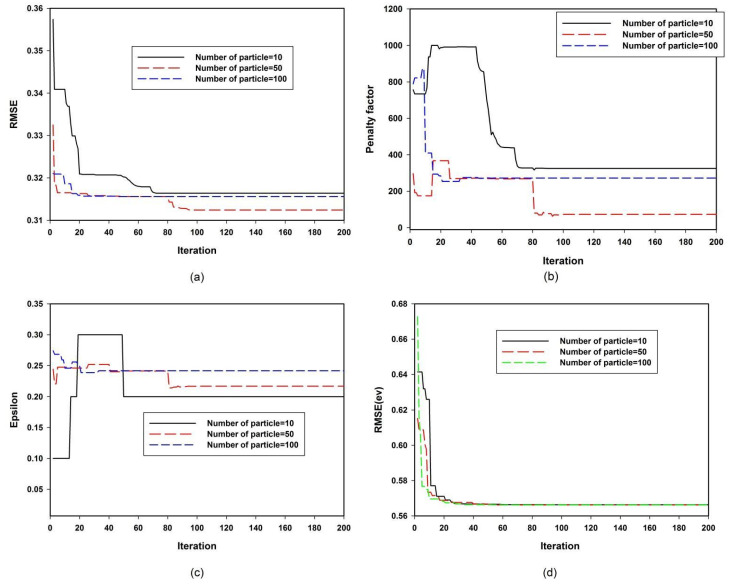

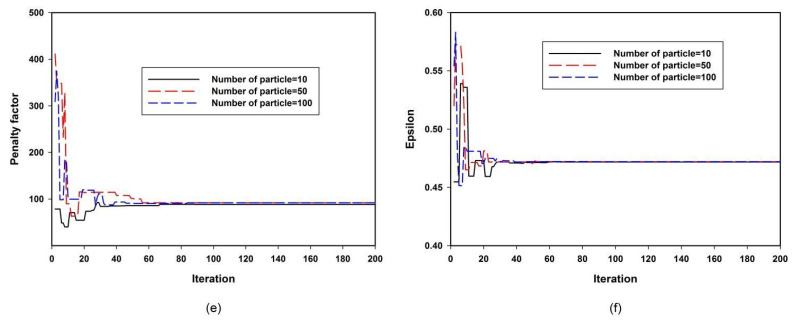
Convergence and sensitivity of the PSVR models. (**a**) Convergence of n-PSVR with various numbers of particles. (**b**) Sensitivity of n-PSVR to the penalty factor. (**c**) Sensitivity of n-PSVR to epsilon. (**d**) Convergence of E-PSVR with various numbers of particles. (**e**) Sensitivity of E-PSVR to the penalty factor. (**f**) Sensitivity of E-PSVR to epsilon.

The influences of the number of swarm particles on the exploration and exploitation capacities of the developed n-PSVR and E-PSVR models are presented in Figure 3. The figure also includes the sensitivity of each of the developed models to the hyperparameters, given various numbers of swarm particles. A balance should be maintained between the exploration and exploitation capacities of the PSO algorithm. When a small number of particles explores a search space, the exploration ability of the algorithm might be hindered. To enhance this exploration capacity by populating the search space with many particles, the exploitation strength of the algorithm might be affected. Figure 3a presents the convergence of the developed n-PSVR model as the number of iterations varies. Premature convergence was observed when the number of particles was set to ten. The figure shows fifty particles in a swarm led to global convergence. The algorithm was trapped in local solutions as the number of particles in the swarm increased from fifty to one hundred. This can be attributed to the deterioration of the exploitation capacity of the algorithm, as the search space was well explored with fifty particles. Figure 3b shows the variation of the penalty factor with the number of particles in the swarm. Less deviation of the estimated refractive index was observed when fifty particles explored the search space. The sensitivity of the developed n-PSVR model to error threshold epsilon is presented in Figure 3c. Although the convergence began at different points when different numbers of swam particles explored the search space, the algorithm converged to the optimum error threshold with fifty particles. This signifies the robustness and precision of the developed model. Error convergence of the developed E-PSVR model is presented in Figure 3d. Irrespective of the number of particles exploiting the search space, the algorithm converged to the same global solution. This shows the robustness of the model we made to have enhanced exploitation and exploration capacities. Figure 3e presents the sensitivity of the E-PSVR model to the penalty factor with different numbers of swarm particles in the search space. The algorithm showed similar global convergence after sixty iterations. Figure 3f shows the sensitivity of the E-PSVR model to the value of error threshold epsilon. The model showed good convergence irrespective of the number of swarm particles. The details of the swarm particles that demonstrated optimum performance, as measured through lowest root mean square error (RMSE), are presented in Table 1. It should be noted that several kernel functions were investigated. The reported Gaussian kernel function showed superior performance over polynomial and sigmoid functions.

### 4.2. Performance Evaluations of the Developed Models

The performance of each of the four developed models was evaluated using error metrics and correlation coefficients. The empirical linear equations for the n-OLR and E-OLR models are presented in Equations (14) and (15), respectively.

(14) $$n-OLR=-0.0628E_{g}+2.1354$$

(15) $$E-OLR=-0.3885n+4.5493_{\;}$$

**Figure 4:**
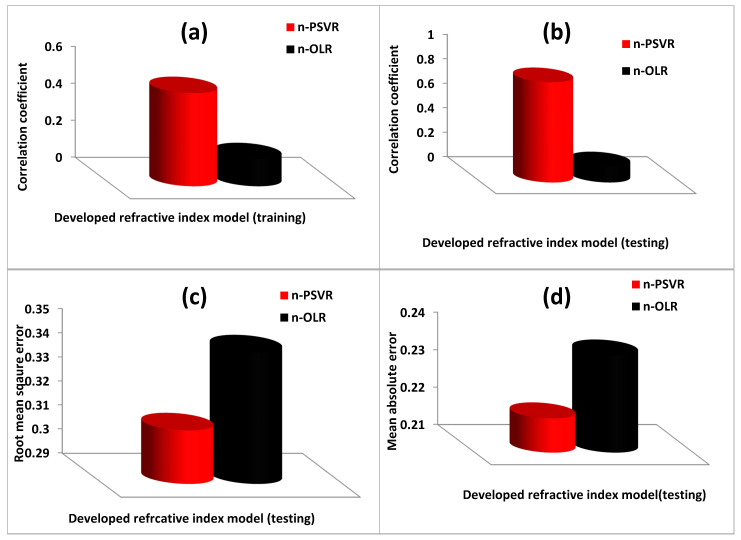
Performance of the developed n-PSVR model using various error metrics. (**a**) Correlation coefficient for each of the developed refractive index model during training phase (**b**) Correlation coefficient for each of the developed refractive index model during testing phase (**c**) Root mean square error for each of the developed refractive index model during training phase (**d**) Mean absolute error for each of the developed refractive index model during testing phase.

The empirical equations were generated using a set of training data and later validated with test data. Evaluations of the performances of n-PSVR and n-OLR models are presented in Figure 4.

The n-PSVR model had superior performance to the n-OLR model in the training and testing stages of model development according to root mean square error (RMSE), mean absolute error (MAE), and correlation coefficient (CC). The n-PSVR model performed better than the n-OLR model during the training phase, as presented in Figure 4a. The performance improvement was 70.83% in terms of CC. The testing phase of model development showed performance improvements of 83.90%, 9.39%, and 7.12% with CC, RMSE, and MAE metrics, respectively, as shown in Figure 4b–d. Performance during the training phase was only compared using CC, since future performance of a model can be effectively judged using the testing performance.

**Figure 5:**
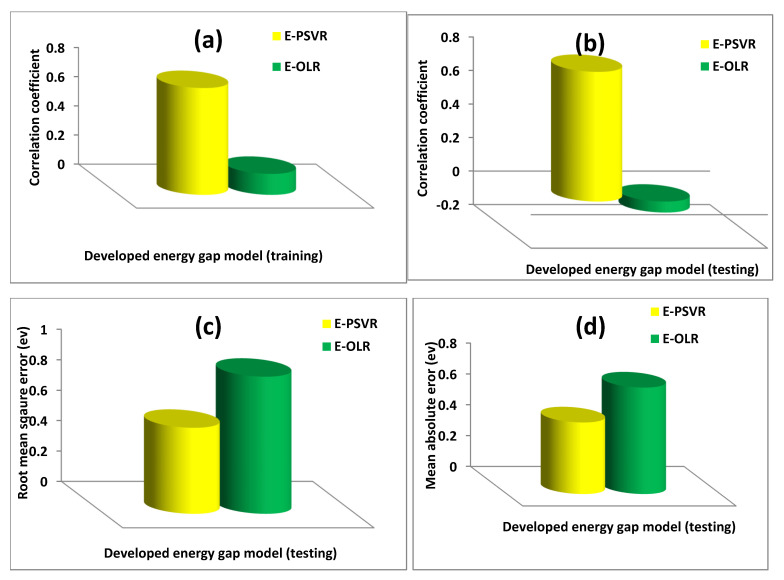
Performance of the developed E-PSVR model using various error metrics. (**a**) Correlation coefficient for each of the developed energy gap model during training phase (**b**) Correlation coefficient for each of the developed energy gap model during testing phase (**c**) Root mean square error for each of the developed energy gap model during training phase (**d**) Mean absolute error for each of the developed energy gap model during testing phase.

The E-PSVR, which can estimate the energy gaps of polyvinyl alcohol composites, performed better than the E-OLR model. The performance enhancement was 80.34% in terms of CC, on training data, as depicted by Figure 5a. Similar performance improvements of 108.46%, 37.28%, and 32.77% in CC, RMSE, and MAE, respectively, were obtained at the testing stage of model development, as presented in Figure 5b–d. The results with all error metrics of the performance evaluation at each stage of model development are presented in Table 2.

### 4.3. The Doping Effect of Sodium-Based Dysprosium Oxide on the Energy Gap of Polyvinyl Alcohol Using E-PSVR

**Figure 6:**
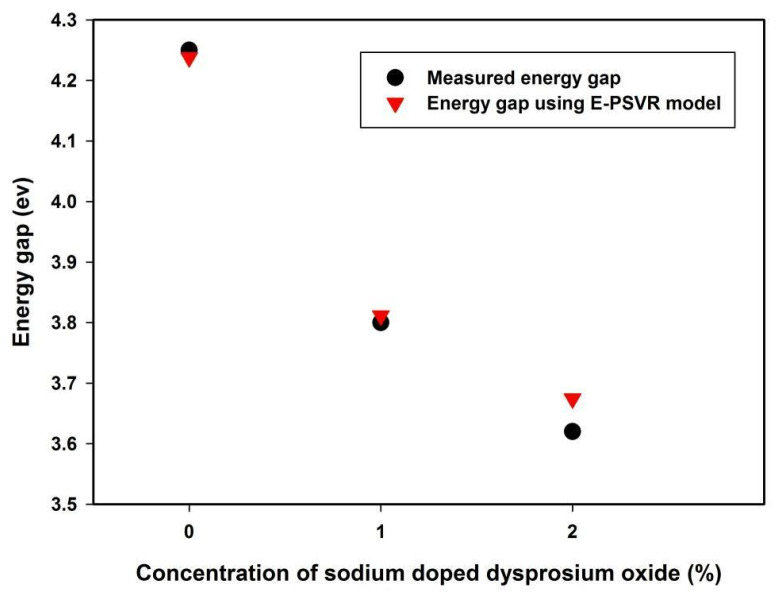
The effect of Na_2_Dy_2_O_4_ dopant on the energy gap of polyvinyl alcohol.

The effect of incorporating sodium-based dysprosium oxide on the energy gap of polyvinyl alcohol using is presented in Figure 6, which was calculated using E-PSVR. The results of the developed E-PSVR model match the measured values well [44].

The gap disjoining the conduction band from the valence band was reduced by the incorporation of the filler (sodium-based dysprosium oxide). This observation can be attributed to the induction of a localized electronic state which facilitated lower-energy electronic transitions [44]. The disorderliness in the doped samples increased as the filler concentration increased, due to structural change in the polymer consequent upon incorporation of the dopant. This experimentally observed energy gap reduction was well captured by the E-PSVR model, except for the sample with a 2% concentration of sodium-based dysprosium oxide, which showed a maximum deviation of 1.5% for the measured and estimated energy gaps of 3.62 and 3.6744 ev, respectively.

### 4.4. The Importance of Benzoxazinone for the Energy Gap of Polyvinyl Alcohol Using the E-PSVR Model

**Figure 7:**
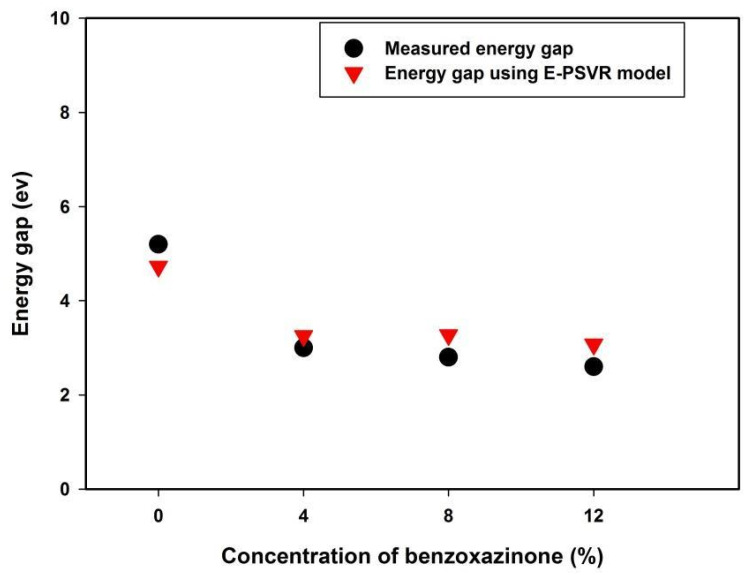
The effect of benzoxazinone’s incorporation on the energy gap of polyvinyl alcohol.

The energy gap lowering effect of incorporating benzoxazinone on polyvinyl alcohol, as obtained by the E-PSVR model, is presented in Figure 7. The figure also presents a comparison between the obtained outcomes of the model and the measured values [6].

The observed energy gap reduction can be attributed to the formation of chemical and structural bonds. The molecules of benzoxazinone each form a bond with polyvinyl alcohol, which enhances the formation of trap levels existing between the lowest unoccupied molecular orbit (LUMO) and highest occupied molecular orbit (HOMO). Therefore, lower energy transitioning becomes feasible, leading to optical energy gap reduction [6].

### 4.5. Further Validation of the E-PSVR and n-PSVR Models Using External Data

To assess the performances of the hybrid E-PSVR and n-PSVR models, external validation was conducted with them. In the validation process, the developed models were only supplied with the model inputs. The models employed the acquired support vectors during the training phase of model development for performing external validation. It should be noted that the external data utilized for the validation process were not included in the training and testing sets of data used for model development. Validation of the n-PSVR model employed thirty-five polyvinyl alcohol composite polymers extracted from different sources. Twenty-eight polyvinyl alcohol composite polymers were used for validating the developed E-PSVR model. Table 3 presents the outcomes of the external validation with the inclusion of percentage error for each of the polyvinyl alcohol composite polymers. The mean absolute percentage errors (MAPE) for the developed n-PSVR and E-PSVR models were 7.92 and 7.57, respectively, for the employed validation data. The standard deviation (SD) of the mean error and the standard error of the mean (SEM) are also presented in the table.

## 5. Conclusions

The optical properties of polyvinyl alcohol composites were modeled in this work using hybrid support vector regression and particle swarm optimization. The results of the hybrid PSVR-based model were compared with the estimates of ordinary linear regression (OLR) models using error metrics such as RMSE, CC, and MAE. The E-PSVR model performed better than the E-OLR model with a performance enhancement of 80.34% in CC on the training data. Similar performance improvements of 108.46%, 37.28%, and 32.77% in CC, RMSE, and MAE, respectively, were obtained at the testing stage of model development. The n-PSVR model also outperformed the n-OLR model using three error metrics. The E-PSVR model was used to investigate the significance of sodium-based dysprosium oxide and benzoxazinone on the energy gap of a polyvinyl alcohol composite. The results agree well with the measured values. The E-PSVR and n-PSVR models were externally validated using thirty-six and twenty-eight polyvinyl alcohol composites, respectively, and the obtained optical properties agree well with the measured values. The outstanding performance demonstrated by these models should strengthen and aid the design of polyvinyl alcohol-based composites for specific industrial and technological applications.

## Figures and Tables

**Table 1:** Details of swarm particles with optimum performance.

	n-PSVR	E-PSVR
C	73.6036	91.937
N_P_	50	50
ε	0.217	0.472
ω	0.2671	0.0587
$\eta \left(E_{k},E\right)$	Gaussian	Gaussian

**Table 2:** Performance evaluations of the developed models.

	Training Phase	Testing Phase		
	CC	CC	RMSE	MAE
n-PSVR	0.5042	0.8196	0.3124	0.2192
n-OLR	0.1470	0.1319	0.3448	0.2360
E-PSVR	0.7332	0.7743	0.5663	0.4645
E-OLR	0.1441	−0.0655	0.9029	0.6909

**Table 3:** Results of external validation of the developed hybrid models.

S/N	Measured Energy Gap (ev)	Measured Refractive Index	n-PSVR	%Error	S/N	Measured Refractive Index	Measured Energy Gap (ev)	E-PSVR (ev)	%Error
1	5.36	1.47 [47]	1.67	13.35	1	2.12	4.27 [48]	4.10	3.89
2	5.06	1.80 [47]	1.82	1.16	2	1.47	5.36 [47]	4.97	7.28
3	2.67	2.01 [47]	2.07	3.14	3	2.01	2.67 [47]	2.82	5.50
4	2.80	1.56 [49]	1.78	14.29	4	2.06	1.82 [47]	1.86	2.20
5	2.40	1.72 [49]	1.59	7.80	5	1.43	5.00 [5]	4.35	13.04
6	3.98	1.67 [50]	1.47	11.68	6	1.44	4.50 [49]	4.53	0.62
7	3.90	1.72 [50]	1.62	5.88	7	1.67	3.98 [50]	3.66	8.03
8	3.81	1.79 [50]	1.78	0.53	8	2.10	3.78 [50]	3.40	9.92
9	3.78	2.10 [50]	1.82	13.42	9	1.94	3.62 [50]	4.04	11.53
10	3.62	1.94 [50]	1.78	8.34	10	1.22	2.70 [51]	2.34	13.46
11	4.96	2.02 [52]	2.14	5.74	11	1.26	2.60 [51]	2.75	5.88
12	5.05	1.99 [52]	1.86	6.65	12	1.54	3.80 [53]	4.36	14.79
13	5.02	2.00 [52]	1.96	1.78	13	2.13	4.20 [54]	4.33	3.20
14	2.40	1.56 [51]	1.59	1.65	14	1.43	4.48 [54]	4.40	1.76
15	2.30	1.65 [51]	1.44	12.61	15	1.53	4.77 [54]	4.30	9.83
16	3.50	1.53 [53]	1.64	7.50	16	1.57	5.20 [55]	4.58	11.98
17	3.60	1.62 [53]	1.75	8.06	17	2.51	4.15 [55]	4.33	4.38
18	4.97	2.00 [54]	2.11	5.65	18	1.91	4.41 [56]	4.03	8.66
19	4.14	1.26 [54]	1.40	11.42	19	1.98	4.32 [56]	3.94	8.80
20	6.45	1.76 [54]	1.55	11.90	20	2.11	4.10 [56]	3.79	7.54
21	5.20	1.57 [54]	1.45	7.83	21	1.41	4.90 [57]	4.20	14.24
22	4.80	2.08 [55]	2.12	1.80	22	2.15	4.60 [57]	4.59	0.29
23	3.10	2.81 [54]	2.96	5.23	23	2.49	4.50 [57]	4.62	2.65
24	5.05	1.99 [54]	1.86	6.84	24	2.89	4.20 [57]	3.78	10.09
25	4.98	2.00 [58]	2.09	4.27	25	1.38	4.76 [59]	4.36	8.47
26	4.91	2.01 [58]	2.21	9.75	26	2.13	4.22 [59]	4.33	2.72
27	4.87	2.02 [58]	2.21	9.49	27	2.67	2.11 [59]	2.38	12.74
28	4.57	1.77 [56]	1.78	0.54	28	2.39	3.01 [60]	3.27	8.51
29	4.41	1.91 [56]	1.75	8.13		SD = 0.19	SEM = 0.04	MAPE =	7.57
30	5.00	1.87 [5]	2.03	8.55					
31	2.56	1.94 [59]	2.13	9.64					
32	3.33	2.31 [60]	2.09	9.60					
33	3.01	2.39 [60]	2.61	9.23					
34	4.61	2.06 [48]	1.80	12.84					
35	4.55	2.07 [48]	1.78	14.14					
36	4.51	2.08 [48]	1.78	14.54					
SD = 0.08		SEM = 0.01	MAPE =	7.92					

## Data Availability

The data supporting the results can be found in literature as stated in Section 3.1 and the additional dataset is also available in Section 4.5 Table 3 of the manuscript.

## Supplementary Materials

The following are available online at https://www.mdpi.com/article/10.3390/polym13162697/s1, The developed source code that implements the proposed hybrid PSVR models is included as supplementary material. The employment data (training, testing and external validation) are also included to ease the reproducibility of the developed models.

## References

[1] Soliman T.S., Vshivkov S.A. (2019). Effect of Fe nanoparticles on the structure and optical properties of polyvinyl alcohol nanocomposite films. J. Non. Cryst. Solids.

[2] Tamgadge Y.S., Talwatkar S.S., Sunatkari A.L., Pahurkar V.G., Muley G.G. (2015). Studies on nonlocal optical nonlinearity of Sr—CuO—polyvinyl alcohol nanocomposite thin films. Thin Solid Films.

[3] Khairy Y., Mohammed M.I., Elsaeedy H.I., Yahia I.S. (2020). Optical and electrical properties of SnBr 2 -doped polyvinyl alcohol (PVA) polymeric solid electrolyte for electronic and optoelectronic applications. Optik.

[4] Devi C.U., Sharma A.K., Rao V.V.R.N. (2002). Electrical and optical properties of pure and silver nitrate-doped polyvinyl alcohol films. Mat. Lett..

[5] Rashad M. (2020). Tuning optical properties of polyvinyl alcohol doped with different metal oxide nanoparticles. Opt. Mater..

[6] El-badry Y.A., Mahmoud K.H. (2019). Molecular and Biomolecular Spectroscopy Optical study of a static benzoxazinone derivative doped poly (vinyl) pyrrolidone—Poly (vinyl) alcohol blend system. Spectrochim. Acta Part A Mol. Biomol. Spectrosc..

[7] Mahmoud K.H., Elsayed K.A., Kayed T.S. (2018). Molecular and Biomolecular Spectroscopy Optical properties of polyvinyl alcohol film irradiated with Nd: YAG laser. Spectrochim. Acta Part A Mol. Biomol. Spectrosc..

[8] Ali H.E., Algarni H., Yahia I.S., Khairy Y. (2021). Optical absorption and linear/nonlinear parameters of polyvinyl alcohol films doped by fullerene. Chin. J. Phys..

[9] Saini I., Rozra J., Chandak N., Aggarwal S., Sharma P.K., Sharma A. (2013). Tailoring of electrical, optical and structural properties of PVA by addition of Ag nanoparticles. Mater. Chem. Phys..

[10] Duchowicz P.R., Fioressi S.E., Bacelo D.E., Saavedra L.M., Toropova A.P., Toropov A.A. (2015). QSPR studies on refractive indices of structurally heterogeneous polymers. Chemom. Intell. Lab. Syst..

[11] Abdelaziz M. (2011). Cerium (III) doping effects on optical and thermal properties of PVA films. Phys. B Phys. Condens. Matter..

[12] Nangia R., Shukla N.K., Sharma A. (2019). Optical and structural properties of Se 80 Te 15 Bi 5/PVA nanocomposite films. J. Mol. Struct..

[13] Ravindra N.M., Ganapathy P., Choi J. (2007). Energy gap–refractive index relations in semiconductors—An overview. Infrared Phys. Technol..

[14] Vapnik V.N. (1998). Statistical Learning Theory.

[15] Vapnik V.N. (1995). The Nature of Statistical Learning Theory.

[16] Basak D., Pal S., Patranabis D.C. (2007). Support Vector Regression. Neural Inf. Process. Lett. Rev..

[17] Owolabi T.O., Abd Rahman M.A. (2021). Prediction of Band Gap Energy of Doped Graphitic Carbon Nitride Using Genetic Algorithm-Based Support Vector Regression and Extreme Learning Machine. Symmetry.

[18] Owolabi T.O., Abd Rahman M.A. (2021). Energy Band Gap Modeling of Doped Bismuth Ferrite Multifunctional Material Using Gravitational Search Algorithm Optimized Support Vector Regression. Crystals.

[19] Olatunji S.O., Owolabi T.O. (2021). Modeling superconducting transition temperature of doped MgB 2 superconductor from structural distortion and ambient temperature resistivity measurement using hybrid intelligent approach. Comput. Mater. Sci..

[20] Tokuyama H., Mori H., Hamaguchi R., Kato G. (2021). Prediction of the lower critical solution temperature of poly(N-isopropylacrylamide-co-methoxy triethyleneglycol acrylate) in aqueous salt solutions using support vector regression. Chem. Eng. Sci..

[21] İskenderoğlu F.C., Baltacioğlu M.K., Demir M.H., Baldinelli A., Barelli L., Bidini G. (2020). Comparison of support vector regression and random forest algorithms for estimating the SOFC output voltage by considering hydrogen flow rates. Int. J. Hydrog. Energy.

[22] Dodangeh E., Panahi M., Rezaie F., Lee S., Tien D. (2020). Novel hybrid intelligence models for flood-susceptibility prediction: Meta optimization of the GMDH and SVR models with the genetic algorithm and harmony search. J. Hydrol..

[23] Beheshti Z. (2020). A time-varying mirrored S-shaped transfer function for binary particle swarm optimization. Inf. Sci..

[24] Wang Y., Li R., Chen Y. (2020). Accurate elemental analysis of alloy samples with high repetition rate laser-ablation spark-induced breakdown spectroscopy coupled with particle swarm optimization-extreme learning machine. Spectrochim. Acta Part B At. Spectrosc..

[25] Adewunmi A.A., Ismail S., Owolabi T.O., Sultan A.S., Olatunji S.O., Ahmad Z. (2019). Hybrid Intelligent Modelling of the Viscoelastic Moduli of Coal Fly Ash Based Polymer Gel System for Water Shutoff Treatment in Oil and Gas Wells. Can. J. Chem. Eng..

[26] Ju X., Liu F., Wang L., Lee W.J. (2019). Wind farm layout optimization based on support vector regression guided genetic algorithm with consideration of participation among landowners. Energy Convers. Manag..

[27] Parsa P., Naderpour H. (2021). Shear strength estimation of reinforced concrete walls using support vector regression improved by Teaching—Learning-based optimization, Particle Swarm optimization, and Harris Hawks Optimization algorithms. J. Build. Eng..

[28] Shamsah S.M.I., Owolabi T.O. (2020). Newtonian mechanics based hybrid machine learning method of characterizing energy band gap of doped zno semiconductor. Chin. J. Phys..

[29] Balogun A., Rezaie F., Bao Q., Gigovi L. (2021). Spatial prediction of landslide susceptibility in western Serbia using hybrid support vector regression (SVR) with GWO, BAT and COA algorithms. Geosci. Front..

[30] Olatunji S.O., Owolabi T.O. (2021). Barium Titanate Semiconductor Band Gap Characterization through Gravitationally Optimized Support Vector Regression and Extreme Learning Machine Computational Methods. Math. Probl. Eng..

[31] Murillo-escobar J., Sepulveda-suescun J.P., Correa M.A., Orrego-metaute D. (2019). Urban Climate Forecasting concentrations of air pollutants using support vector regression improved with particle swarm optimization: Case study in Aburrá Valley, Colombia. Urban Clim..

[32] Rui J., Zhang H., Zhang D., Han F., Guo Q. (2019). Journal of Petroleum Science and Engineering Total organic carbon content prediction based on support-vector-regression machine with particle swarm optimization. J. Pet. Sci. Eng..

[33] Liu M., Luo K., Zhang J., Chen S. (2021). A stock selection algorithm hybridizing grey wolf optimizer and support vector regression. Expert Syst. Appl..

[34] Owolabi T.O. (2019). Development of a particle swarm optimization based support vector regression model for titanium dioxide band gap characterization. J. Semicond..

[35] Zhang L., Zhao L. (2021). High-quality face image generation using particle swarm optimization-based generative adversarial networks. Futur. Gener. Comput. Syst..

[36] Akande K.O., Owolabi T.O., Olatunji S.O., AbdulRaheem A. (2016). A hybrid particle swarm optimization and support vector regression model for modelling permeability prediction of hydrocarbon reservoir. J. Pet. Sci. Eng..

[37] Aziz S., Nofal M., Ghareeb H., Dannoun E., Hussen S., Hadi J., Ahmed K., Hussein A. (2021). Characteristics of poly(Vinyl alcohol) (PVA) based composites integrated with green synthesized Al3+-metal complex: Structural, optical, and localized density of state analysis. Polymers.

[38] Dhatarwal P., Sengwa R.J. (2021). Investigation on the optical properties of (PVP/PVA)/Al2O3 nanocomposite films for green disposable optoelectronics. Phys. B Condens. Matter..

[39] Ali F.M. (2019). Structural and optical characterization of [(PVA:PVP)-Cu2+] composite films for promising semiconducting polymer devices. J. Mol. Struct..

[40] Ismail A.M., Mohammed M.I., Yahia I.S. (2020). A facile method to prepare g-carbon nitride/poly (vinyl alcohol) nanocomposite films with remarkable optoelectrical properties: Laser attenuation approach. Opt. Laser Technol..

[41] Ali F.M., Kershi R.M., Sayed M.A., Aboudeif Y.M. (2018). Physica B: Condensed Matter Evaluation of structural and optical properties of Ce 3 þ ions doped (PVA/PVP) composite fi lms for new organic semiconductors. Phys. B Phys. Condens. Matter..

[42] Mahmoud K.H. (2015). Molecular and Biomolecular Spectroscopy Synthesis, characterization, optical and antimicrobial studies of polyvinyl alcohol—Silver nanocomposites. Spectrochim. ACTA PART A Mol. Biomol. Spectrosc..

[43] Alibwaini Y.A., Hemeda O.M., El-shater R., Sharshar T., Ashour A.H. (2020). Synthesis, characterizations, optical and photoluminescence properties of polymer blend PVA/PEG films doped eosin Y (EY) dye. Opt. Mater..

[44] Shilpa K.N., Subramani K., Sachhidananda S., Madhukar B.S. (2017). Visibly transparent PVA/sodium doped dysprosia (Na 2 Dy 2 O 4) nano composite films, with high refractive index: An optical study. J. Alloys Compd..

[45] Ali H.E., Khairy Y. (2019). Condensed Matter Microstructure and optical properties of Ni_2_ + doped PVA for optoelectronic devices. Phys. B Phys. Condens. Matter..

[46] Ghanipour M., Dorranian D. (2013). Effect of Ag-Nanoparticles Doped in Polyvinyl Alcohol on the Structural and Optical Properties of PVA Films. J. Nanomater..

[47] Yahia I.S., Mohammed M.I., Nawar A.M. (2019). Multifunction applications of TiO_2_/poly(vinyl alcohol) nanocomposites for laser attenuation applications. Phys. B Condens. Matter..

[48] Morsi M.A., Asnag G.M., Rajeh A., Awwad N.S. (2021). Nd:YAG nanosecond laser induced growth of Au nanoparticles within CMC/PVA matrix: Multifunctional nanocomposites with tunable optical and electrical properties. Compos. Commun..

[49] Donya H., Taha T.A., Alruwaili A., Tomsah I.B.I., Ibrahim M. (2020). Micro-structure and optical spectroscopy of PVA/iron oxide polymer nanocomposites. J. Mater. Res. Technol..

[50] Arandhara G., Mostako A.T.T., Dutta P., Bora J., Saikia P.K. (2020). Influence of thermolysis temperature on the morphology, structural and optical properties of nanocomposite ZnS-polyvinyl alcohol thin films: Fabrication and characterization of indium tin oxide/ZnS-polyvinyl alcohol/Al Schottky diode. Thin Solid Films.

[51] Imam N.G., Mohamed M.B. (2016). Environmentally friendly Zn0.75Cd0.25S/PVA heterosystem nanocomposite: UV-stimulated emission and absorption spectra. J. Mol. Struct..

[52] Awwad N.S., El-Kader M.F.H.A., Ibrahium H.A., Asnag G.M., Morsi M.A. (2020). Green synthesis of different ratios from bimetallic gold: Silver nanoparticles core@shell via laser ablation scattered in Chitosan-PVA matrix and its electrical conductivity behavior. Compos. Commun..

[53] Heiba Z.K., Mohamed M.B., Imam N.G. (2017). Fine-tune optical absorption and light emitting behavior of the CdS/PVA hybridized film nanocomposite. J. Mol. Struct..

[54] Menazea A.A., Ismail A.M., Awwad N.S., Ibrahium H.A. (2020). Physical characterization and antibacterial activity of PVA/Chitosan matrix doped by selenium nanoparticles prepared via one-pot laser ablation route. J. Mater. Res. Technol..

[55] Kavya H.V., Nithin K.S., Kendagannaswamy B.K., Sachhidananda S., Chamaraja N.A. (2020). Optical performance appraisal of mechanically flexible and visibly clear PVP-PVA/calcium doped zirconium oxide nanocomposites for UV shielding applications. Optik.

[56] Chahal R.P., Mahendia S., Tomar A.K., Kumar S. (2015). UV irradiated PVA-Ag nanocomposites for optical applications. Appl. Surf. Sci..

[57] Soliman T.S., Zaki M.F., Hessien M.M., Elkalashy S.I. (2020). The structure and optical properties of PVA-BaTiO3 nanocomposite films. Opt. Mater..

[58] Choudhary S., Sengwa R.J. (2018). ZnO nanoparticles dispersed PVA–PVP blend matrix based high performance flexible nanodielectrics for multifunctional microelectronic devices. Curr. Appl. Phys..

[59] Chandrappa H., Bhajantri R.F., Prarthana N. (2020). Simple fabrication of PVA-ATE (Amaranthus tricolor leaves extract) polymer biocomposites: An efficient UV-Shielding material for organisms in terrestrial and aquatic ecosystems. Opt. Mater..

[60] Elashmawi I.S., Menazea A.A. (2019). Different time’s Nd:YAG laser-irradiated PVA/Ag nanocomposites: Structural, optical, and electrical characterization. J. Mater. Res. Technol..

